# In Vitro Protective Effect of Paste and Sauce Extract Made with *Protaetia brevitarsis* Larvae on HepG2 Cells Damaged by Ethanol

**DOI:** 10.3390/insects11080494

**Published:** 2020-08-03

**Authors:** Dooseon Hwang, Tae-Won Goo, Eun-Young Yun

**Affiliations:** 1 Department of Integrative Biological Sciences and Industry, Sejong University, Seoul 05006, Korea; h.michael8837@gmail.com; 2 Department of Biochemistry, School of Medicine, Dongguk University, Gyeongju, Kyeongbuk 38066, Korea; gootw@dongguk.ac.kr

**Keywords:** *Protaetia brevitarsis*, paste, sauce, hepatoprotective

## Abstract

We made paste and sauce using protein-rich *Protaetia brevitarsis* larvae (PBL) and evaluated their fermentation levels. After pretreatment with the paste and sauce extracts, HepG2 cells were damaged with ethanol (EtOH), and then the effects of the paste and sauce were evaluated. As a result, we confirmed that the PBL paste and sauce extracts reduced the aspartate aminotransferase (AST) and alanine aminotransaminase (ALT) content in the medium as compared to soybean (*Glycine max*) sauce and paste extracts. In addition, the PBL paste and sauce extracts significantly lowered the level of tumor necrosis factor (TNF)-α and interleukin (IL)-6, which are biomarkers of inflammation, and significantly increased the inhibition rate of superoxide dismutase (SOD) and reduced glutathione (GSH), which are antioxidative indicators, in proportion to the amount of PBL added to the paste and sauce. These results suggest that an intake of PBL paste and sauce, a novel type of fermented food made from insects, may be effective for liver protection through anti-inflammatory and antioxidative effects against hepatocyte injury caused by EtOH.

## 1. Introduction

Insects are emerging as a new protein-rich food source because of the many advantages they possess as a protein food [1]. They are abundant and diverse enough to make up more than 70% of the animals on the planet, have excellent feed efficacy, are eco-friendly because they hardly emit greenhouse gases during breeding, and do not require high skill standards in breeding [2]. Asian countries have used insects as food for a long time. In particular, there are records to show that insects have been used in China as a food source since approximately 3200 years ago [3]. In Korea, *Protaetia brevitarsis* larvae (PBL), *Tenebrio molitor* larvae (TML), *Allomyrina dichotoma* larvae, *Zophobas morio* larvae, *Gryllis bimaculatus*, *Bombyx mori* larvae and pupae, and *Oxya chinensis sinuosa* can now be legally used as edible insects. Accordingly, studies have been actively conducted in Korea to develop various types of food using insects.

*P. brevitarsis* is an insect belonging to the Coleoptera Scarabaeidae family. It has already been proven as non-toxic through good laboratory practice (GLP) procedures [4,5] and reported to consist of, among others, 58% protein, 10% carbohydrate, and 16% fat [6]. PBL is known to contain useful bioactive substances, such as protacin—a natural antibiotic protein. It has also been reported that ALT, bilirubin, and bile acid levels are restored to normal levels when orally administering PBL to carbon-tetrachloride-induced rats [7]. *P. brevitarsis* has been used for a long time in Korea as a traditional medicine for liver disease, as its efficacy on the liver had been recorded in the old Korean medical book entitled Donguibogam in 1613 [8]. On the other hand, PBL extract has been found to include anticancer substances composed of at least three fatty acids and can cause tumor cell death through apoptosis induced by caspase-3 activation in tumor cells [9].

Soybean (*Glycine max*) sauce (Ganjang) and paste (Doenjang) are made from soybean koji, which is fermented by inoculating microorganisms or inducing the natural attachment of microorganisms. Fermented soybean koji is aged in salt water for 2–3 months, and the liquid and solid are separated from each other. The liquid is Ganjang, and the solid is Doenjang [10]. During maturation, their starch, fat, and protein are degraded into free sugars, organic acids, and free amino acids, respectively, by microorganisms to help digestion in the body. As Ganjang and Doenjang have a unique aroma and flavor in addition to a salty taste [11], they have been used in Korea as traditional seasoning. Similar to other fermented foods, such as Kimchi [12] and cheese [13], they have been reported to have diverse functionalities, such as anticancer, antioxidant, thrombolytic, hypoglycemic, and other adult disease suppressing activities [9,14,15,16,17,18,19]. 

In addition to using soybean as a vegetable protein source, many researchers have developed fermented foods using other food ingredients, such as animal protein sources, and conducted functional studies for them. Previous studies have shown that fermented shrimp sauce increases antioxidant activity as the fermentation time increases [20]. In addition, an oxygen radical absorbance capacity experiment with fish sauce, which is another fermented animal protein food, showed that the sauce has more antioxidant activity than apples, limes, oranges, pears, and white grapes, which have previously been reported to have high antioxidant activity [19]. Previous studies have reported the sensory evaluation and physicochemical activities of TML sauce prepared with an inoculation of *Aspergillus oryzae* and *Bacillus licheniformis* [21] and the protective activity of TML fermented foods against ethanol (EtOH)-damaged HepG2 cells [22]. However, there have been no reports of fermented foods using PBL.

In this study, we evaluated the fermentation degree of PBL koji fermented by inoculation of *A. oryzae* in protein-rich (57.86% by dry weight basis) PBL, which are registered as edible insects and a type of new food raw material in Korea [6]. We also assessed the fermentation degree of PBL sauce and paste manufactured from PBL koji. As part of this functional study, we investigated the effect of PBL on alcoholic liver cells that could develop acute liver damage and alcoholic hepatitis accompanied by symptoms such as cirrhosis, fibrosis, steatosis, and steatohepatitis [23]. 

## 2. Materials and Methods

### 2.1. Preparation of PBL Koji, Paste, and Sauce

The soybean (Baektae, 2017 cultivate year) was cropped in Sunchang, Korea, and *A. oryzae* was bought from Chungmu Fermentation (Ulsan, Korea). The soybeans were immersed in distilled water for 12 h and braised for 1.5 h. For the preparation of koji, the steamed PBL and soybeans were used alone or mixed at a ratio of 3:7 or 1:1, mashed, and inoculated with 0.2%, 0.4%, and 0.8% *A. oryzae*. Afterward, the PBL and/or soybeans were molded into a square frame and fermented for 72 h at 30 °C, with a relative humidity of 70%. After fermentation, the samples were dehydrated in a dry oven for 24 h at 60 °C, completing the koji preparation. Subsequently, the koji was soaked in 20% salt water for 50 days. After 50 days, the paste (solid) and sauce (liquid) were separated, and then the sauce was sterilized at 110 °C. Depending on the proportion of PBL added into the koji, we made PBP100 (100% PBL paste), PBP50 (50% PBL/50% *Glycine max* (GM)), and PBP30 (30% PBL/70% GM) pastes and PBS100 (100% PBL sauce), PBS50 (50% PBL/50% GM), and PBS30 (30% PBL/70% GM) sauces for this study. The GMP100 (100% GM paste) and GMS100 (100% GM sauce) used in this study were purchased from SEMPIO, Seoul, Korea.

### 2.2. Preparation of Koji, Paste, and Sauce Extracts

The koji prepared in this study was milled and diluted with distilled water at a ratio of 1:10 (*w/v*). The diluted samples were mixed for 1 h using a shaker, centrifuged at 4000 rpm for 10 min, and then filtered using Whatman^®^ No.1 filter paper for primary filtration and a 0.45 μm syringe filter for secondary filtration.

The paste made from koji was freeze-dried, milled, diluted with 95% EtOH at a ratio of 1:5 (*w/v*), and extracted over 24 h. After centrifugation at 3000 rpm for 10 min, the supernatant was filtered using Whatman^®^ No.1 filter paper for primary filtration and a 0.45 μm syringe filter for secondary filtration. Afterward, the supernatant was concentrated using a speed vacuum (EYELA, Japan) for 48 h and dissolved in 20% DMSO. The sauce was centrifuged at 3000 rpm for 10 min and filtered using Whatman^®^ No.1 filter paper for primary filtration and a 0.45 μm syringe filter for secondary filtration. Each koji, paste, and sauce extract was applied to all the experiments in this study.

### 2.3. Amino Nitrogen, Protease Activity, and Free Amino Acid Analyses

A 95% EtOH extract of koji, paste, and sauce was used in the amino nitrogen and protease activity assays. Amino nitrogen was determined using a Primary Amino Nitrogen Kit (Megazyme, Chicago, IL, USA), and protease activity was determined using a Pierce™ Protease Assay Kit (Thermofisher, Tewksbury, MA, USA), according to each supplier’s protocol. The extracted samples were analyzed for free amino acids, according to the method of the Korean Food Standard Codex (Ministry of Food and Drug Safety, 2019). The prepared samples were blended with 16% trichloroacetate and rocked for 15 min. After centrifugation at 3000 rpm for 15 min, the analysis of free amino acids by liquid chromatography (Agilent, Santa Clara, CA, USA) was performed with the supernatant. 

### 2.4. Cell Culture, EtOH-Damaged HepG2 Cells, and Cell Viability Assay

HepG2 cells were incubated at 37 °C in a 5% carbon dioxide atmosphere with DMEM (LM001-11, WELGENE, Seoul, Korea) containing 1000 mg/L glucose, 10% FBS, and 1% antibiotic–antimycotic (AA) (Life Technology, Carlsbad, CA, USA). When the cell saturation reached 80% confluence, the cells were suspended in trypsin, plated at 1 × 10^6^ cells per well of a 6-well plate, and incubated for 24 h. Afterward, the media were exchanged with 2 mL of DMEM containing 10% FBS, 1% AA, and the paste or sauce extract. After incubation for 4 h, the media were changed with 2 mL DMEM containing 10% FBS, 1% AA, and 300 mM EtOH. Each well was covered with parafilm to prevent EtOH vaporization and incubated for 24 h. After incubation, the cells and media were divided by centrifugation (3000 rpm, 5 min) and applied to all the experiments in this study. HepG2 cell viability was measured using a CellTiter 96^®^ AQ_ueous_ One Solution Cell Proliferation Assay Kit (3-(4,5-dimethylthiazol-2-yl)-5-(3-carboxymethoxyphenyl)-2-(4-sulfophenyl)-2H-tetrazolium, inner salt; MTS; Promega, Madison, WI, USA). 

### 2.5. Aspartate Aminotransferase (AST) and Alanine Aminotransaminase (ALT) Analyses

The media isolated in Section 2.4 were used for AST and ALT analyses. The levels of AST and ALT were measured using Asan GOT (Glutamic oxalacetic transaminase) and GPT (Glutamic pyruvate transaminase) (ASAN Pharmaceutical, Hwaseong, Korea), respectively, according to the supplier’s manuals. Silymarin was used as a positive control. Silymarin has been reported to exert an antioxidant effect by removing oxygen free radicals and reducing lipid peroxidation, thereby promoting hepatocyte regeneration [24,25,26]. The AST and ALT levels are expressed in IU/L.

### 2.6. Total RNA Extraction and Quantitative PCR Analysis 

The total RNA of the HepG2 cells was isolated using TRIzol^®^ Reagent (Ambion, Naugatuck, CA, USA), and cDNA was synthesized using a High Capacity cDNA Reverse Transcription Kit (Applied Biosystems, Foster City, CA, USA). TNF-α and IL-6 levels were measured by the BrightGreen 2X qPCR MasterMix-No Dye (Applied Biological Materials, Richmond, BC, Canada), according to the supplier’s manual. Glyceraldehyde 3-phosphate dehydrogenase (GAPDH) was used as an endogenous control, and relative gene expression levels were analyzed using the 2^−ΔΔCt^ method. Primers for GAPDH (forward 5′-ACCCACTCCTCCACCTTTGA-3′, reverse 5′-CTGTTGCTGTAGCCAAATTCGT-3′), TNF-α (forward 5′-GGAGAAGGGTGACCGACTCA-3′, reverse 5′-CTGCCCAGACTCGGCAA-3′), and IL-6 (forward 5′-GGAGACTTGCCTGGTGAAAA, reverse 5′-GTCAGGGGTGGTTATTGCAT-3′) were used for cDNA amplification [27].

### 2.7. Glutathione (GSH) Activity and Superoxide Dismutase (SOD) Inhibition Rate Analyses

The cells isolated in Section 2.4 were lysed with a radioimmunoprecipitation assay (RIPA) buffer (Biosesang, Korea) and used for the GSH activity and SOD inhibition rate analyses. The GSH activity was analyzed using a Glutathione Assay Kit (Sigma-Aldrich, St. Louis, MO, USA), and the SOD activity (inhibition rate (%)) was analyzed using a SOD Assay Kit (Sigma-Aldrich), according to the manufacturer’s protocols. Silymarin was used as a positive control. The GSH activity and the SOD inhibition rate are represented as relative values of the samples for the non-treatment control.

### 2.8. Statistical Analysis

The results are indicated as the mean ± standard deviation (SD), and all measurements were repeated three times. Differences between groups were tested with the Student’s *t*-test. SPSS version 18 (SPSS Inc., Chicago, IL, USA) was used for analysis. Statistical significance was defined at 0.05 probability.

## 3. Results

### 3.1. Evaluating the Fermentation of Koji Made with PBL

**Figure 1:**
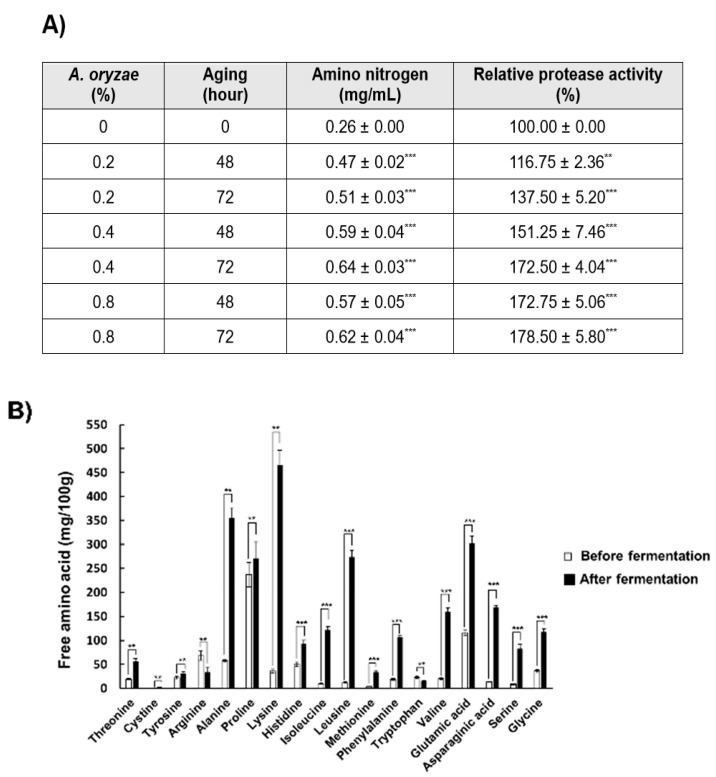
Evaluation of the fermentation of koji made with PBL. Quantitative analyses of the amino nitrogen content and protease activity (**A**) and the free amino acid content (**B**) of PBL koji were performed. Values are the means ± standard deviations of triplicate experiments. ** *p* < 0.01 and *** *p* < 0.001 indicate a significant difference between non-aging and aging treatments.

Amino nitrogen content, protease activity, and free amino acid content were measured to evaluate the degree of PBL fermentation by the inoculation amount of *A. oryzae* and fermentation time. The amino nitrogen content and protease activity increased in proportion to the quantity of *A. oryzae* inoculum and fermentation time. The greatest value of 0.64 mg/mL was measured when the PBL koji had been fermented for 72 h after inoculation with 0.4% *A. oryzae* (Figure 1A). For the protease activity, the maximum value of 178.5% compared with the control (non-fermented PBL) was also observed when the PBL koji had been fermented for 72 h after inoculation with 0.8% *A. oryzae* (Figure 1A). As a result of the free amino acid content analysis before and after fermentation, all the free amino acids except arginine and tryptophan increased after fermentation in this study (Figure 1B). Therefore, PBL pastes and sauces were made using the PBL koji fermented with 0.8% *A. oryzae* for 72 h. Using this fermentation condition, PBL pastes and sauces using koji were made with different proportions of PBL and soybean: GMP100 (100% GM paste), PBP100 (100% PBL paste), PBP50 (50% PBL/50% GM), and PBP30 (30% PBL/70% GM) as pastes and GMS100 (100% GM sauce), PBS100 (100% PBL sauce), PBS50 (50% PBL/50% GM), and PBS30 (30% PBL/70% GM) as sauces.

### 3.2. Evaluation of Aging on Paste and Sauce Made with PBL Koji 

To evaluate the degree of aging in the PBL pastes and sauces made with PBL koji, the amino nitrogen and protease activity were measured in each PBL paste and sauce (Table 1). The amino nitrogen content and protease activity of the paste increased in proportion to the PBL concentration in the koji. For the paste, the maximum values of amino nitrogen (0.45 mg/mL) and protease activity (129.10%) were shown in PBP100. In addition, for the sauce, the amino nitrogen content and protease activity increased with the PBL in a concentration-dependent manner. The maximum values of amino nitrogen (3.88 mg/mL) and protease activity (133.53%) were measured in PBS100. Accordingly, it was found that the PBL pastes and sauces were effectively aged in proportion to the PBL content of the koji.

### 3.3. EtOH Concentration Causing Toxicity to Hepatocytes and Toxicity Assessment of PBL Paste and Sauce to Hepatocytes

**Figure 2:**
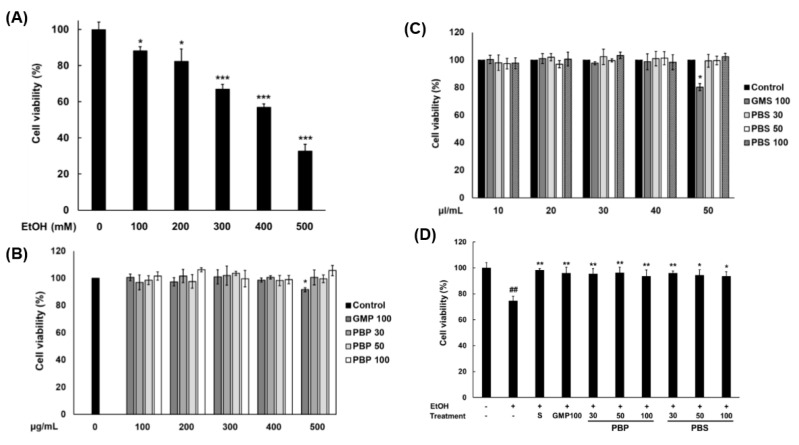
HepG2 cell viability. (**A**) Viability of HepG2 cells treated with 0, 100, 200, 300, 400, and 500 mM EtOH. Values are the means ± standard deviations of triplicate experiments. * *p* < 0.05 and *** *p* < 0.001 indicate a significant difference between the 0 mM and the 100, 200, 300, 400, and 500 mM EtOH. (**B**,**C**) show the cell viability after 300 mM EtOH treatment in the HepG2 cells pretreated with the paste and sauce extracts, respectively, by concentration. Values are the means ± standard deviations of triplicate experiments. * *p* < 0.05 indicates a significant difference between the control and 500 μg/mL of the GMP100 treatment. (**D**) Cell viability after 300 mM EtOH treatment in the HepG2 cells pretreated with the paste and sauce extracts. Values are the means ± standard deviations of triplicate experiments. ## *p* < 0.01 indicates a significant difference between the EtOH (−)/treatment (−) and the EtOH (+)/treatment (−). * *p* < 0.05 and ** *p* < 0.01, indicate a significant difference between the EtOH (+)/treatment (−) and the EtOH (+)/treatment (+). S, silymarin (100 μg/mL); GMP100, paste made from 100% GM; PBP30, paste made from 30% PBL/70% GM; PBP50, paste made from 50% PBL/50% GM; PBP100, paste made from 100% PBL; GMS100, sauce made from 100% GM; PBS30, sauce made from 30% PBL/70% GM; PBS50, sauce made from 50% PBL/50% GM; PBS100, sauce made from 100% PBL. The concentrations of paste and sauce were 400 μg/mL and 40 μl/mL, respectively. Values are the means ± standard deviations of triplicate experiments.

In order to determine the concentration of EtOH that can damage HepG2 cells, the cell viability was measured after adding EtOH at different concentrations. As a result, the viabilities of the HepG2 cells treated with 100, 200, 300, 400, and 500 mM EtOH were 88%, 85%, 67%, 57%, and 33%, respectively. Since a significant difference of *p <* 0.001 was shown in the 300 mM EtOH concentration, 300 mM was determined as the concentration of EtOH that causes toxicity to hepatocytes (Figure 2A). 

In order to evaluate the toxicity of the paste and sauce, the extracts were treated with different concentrations in HepG2 cells. For the paste extract, cell viability decreased at 500 μg/mL of GMP100, and for the sauce extract, cell viability decreased at 50 μl/mL of GMS100 (Figure 2B,C). Accordingly, it was found that the maximum concentrations that did not cause toxicity to the cells of the pastes and sauces used in this study were 400 μg/mL and 40 μl/mL, respectively. 

To evaluate the effect of the PBL paste and sauce on hepatocytes damaged by EtOH, the HepG2 cells were treated with the determined concentration of the PBL paste and sauce extracts (Figure 2D). After treating with 300 mM EtOH, the cells were incubated for 24 h, and then the cell viability was examined. The cell viability of the group treated with only 300 mM EtOH, without the paste or sauce extract, was 75.13%. The viability of cells pretreated with silymarin (positive control), GMP100, PBP30, PBP50, PBP100, PBS30, PBS50, and PBS100 were 98.43%, 96.84%, 95.68%, 94.99%, 96.18%, 94.85%, and 94.57%, respectively. Considering the above results, it can be stated that all the PBL pastes and sauces used in this study effectively protect HepG2 cells against alcohol damage. 

### 3.4. Hepatoprotective Effect of Pretreatment with PBL Paste and Sauce on EtOH-Damaged Hepatocytes 

The AST and ALT levels were measured to evaluate the hepatoprotective potential of PBL paste and sauce in this study (Table 2). First, we proved that the AST and ALT levels in HepG2 cells damaged by EtOH were 56.45 IU/L and 34.46 IU/L, respectively, and that they significantly rose compared to those without EtOH treatment (23.23 IU/L and 25.67 IU/L, respectively). Then, we determined whether pretreating with PBL paste and sauce extract before the EtOH treatment significantly decreased the AST and ALT levels in HepG2 cells as occurred with the silymarin pretreatment (23.35 IU/L and 18.24 IU/L, respectively), which was the positive control.

The AST levels in the EtOH-injured HepG2 cells after pretreatment with GMP100, PBP30, PBP50, and PBP100 significantly decreased compared to only EtOH-treated HepG2 cells (56.45 IU/L). The AST levels in the cells pretreated with GMP100, PBP30, PBP50, and PBP100 extracts were 36.11, 37.35, 29.69, and 25.26 IU/L, respectively. For the pretreatment with GMS100, PBS30, PBS50, and PBS100 extracts, the AST levels were 36.78, 35.18, 33.47, and 28.23 IU/L, respectively. Accordingly, pretreatment with GMS100, PBP30, PBP50, PBP100, PBS30, PBS50, and PBS100 extracts significantly decreased the AST levels in EtOH-damaged HepG2 cells compared to only EtOH-treated HepG2 cells. In addition, compared to the single substance, silymarin (23.32 IU/L), a similar decrease was observed for all treatments (*p* < 0.01). The ALT levels in the EtOH-damaged HepG2 cells after pretreatment with PBP30, 50, and 100 extracts were significantly decreased compared with only EtOH-treated HepG2 cells (34.46 IU/L). The ALT levels in the cells pretreated with GMP100, PBP30, PBP50, and PBP100 extracts were 24.59, 27.62, 21.27, and 19.75 IU/L, respectively. Likewise, for the pretreatment with GMS100, PBS30, PBS50, and PBS100 extracts, the ALT levels were 26.43, 25.66, 20.55, and 19.22 IU/L, respectively. These results showed that the AST and ALT levels in the EtOH-injured HepG2 cells after pretreatment with PBL paste and sauce were lower than those after treatment with GM paste and sauce, except PBP30. The ALT levels showed a decrease similar to that of silymarin (18.24 IU/L) when the larvae contents were 50% and 100%. As the content of insects increased in the paste and sauce, it was found that the AST and ALT levels decreased. In particular, it was confirmed that the reduction effect was highest in the paste and sauce made with 100% PBL. Although the crude extract of the paste and sauce was used, the hepatoprotective activity was similar to that of silymarin, a single substance. In addition, when the content of insects was more than 50%, the AST and ALT reduction effects were greater than those of the GM-based paste and sauce.

### 3.5. Evaluation of Anti-Inflammatory Potential of PBL Paste and Sauce in EtOH-Damaged Hepatocytes

**Figure 3:**
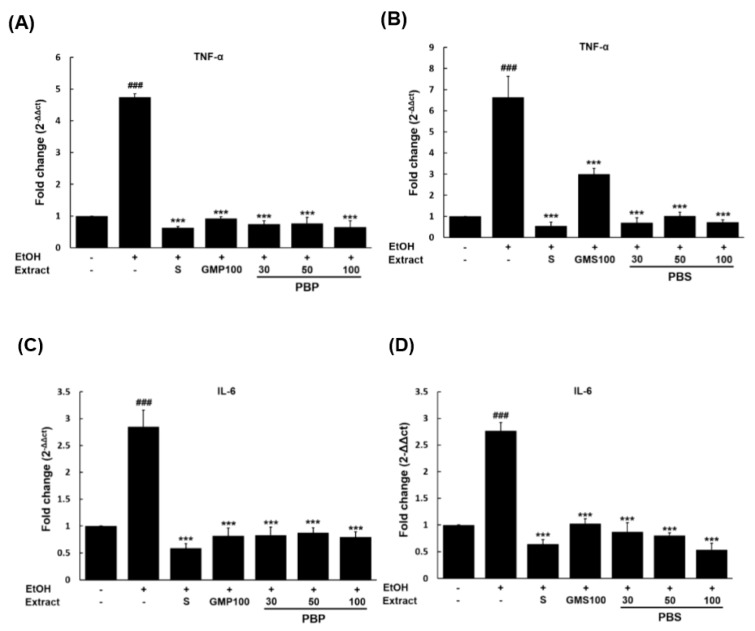
Anti-inflammatory potential of the paste and sauce made from PBL koji. TNF-α levels were analyzed after 300 mM EtOH treatment on HepG2 cells pretreated with 95% EtOH extract of each paste (**A**) and sauce (**B**). IL-6 levels were measured after 300 mM EtOH treatment on HepG2 cells pretreated with 95% EtOH extract of each paste (**C**) and sauce (**D**). S, silymarin (100 μg/mL); GMP100, paste made from 100% GM; PBP30, paste made from 30% PBL/70% GM; PBP50, paste made from 50% PBL/50% GM; PBP100, paste made from 100% PBL; GMS100, sauce made from 100% GM; PBS30, sauce made from 30% PBL/70% GM; PBS50, sauce made from 50% PBL/50% GM; PBS100, sauce made from 100% PBL. The concentrations of the paste and sauce were 400 μg/mL and 40 μl/mL, respectively. Values are the means ± standard deviations of triplicate experiments. ### *p* < 0.001 indicates a significant difference between the EtOH (−)/treatment (−) and the EtOH (+)/treatment (−). *** *p* < 0.001 indicates a significant difference between the EtOH (+)/treatment (−) and the EtOH (+)/treatment (+).

To investigate the anti-inflammatory potential of PBL paste and sauce on EtOH-damaged hepatocytes, the expression levels of TNF-α and IL-6 mRNA were measured in HepG2 cells treated with 300 mM EtOH after pretreatment with PBL paste and sauce extract (Figure 3). First, it was proven that the expression levels of TNF-α and IL-6 transcripts, which are biomarkers of inflammation, were significantly increased in EtOH-treated HepG2 cells compared to normal HepG2 cells (EtOH (−)/treatment (−)). The expression levels of TNF-α in the EtOH-injured HepG2 cells after pretreatment with silymarin, GMP100, PBP30, PBP50, and PBP100 extracts were 0.63-, 0.92-, 0.74-, 0.77-, and 0.65-fold, respectively, compared to the EtOH-treatment control (4.74-fold) (Figure 3A). On the other hand, for the pretreatment with silymarin, GMS100, PBS30, PBS50, and PBS100 extracts, the expression levels of TNF-α were 0.55-, 2.99-, 0.69-, 1.01-, and 0.72-fold, respectively, compared to the EtOH-treatment control (6.64-fold) (Figure 3B). The expression levels of IL-6 in the EtOH-injured HepG2 cells after silymarin, GMP100, PBP30, PBP50, and PBP100 treatment were 0.59-, 0.82-, 0.83-, 0.87-, and 0.79-fold, respectively, compared to the EtOH-treatment control (2.85-fold) (Figure 3C). Likewise, for the pretreatment with silymarin, GMS100, PBS30, PBS50, and PBS100, the expression levels of IL-6 were 0.64-, 1.03-, 0.87-, 0.80-, and 0.54-fold, respectively, compared to the EtOH-treatment control (2.77-fold) (Figure 3D). Based on the above results, the expression levels of TNF-α and IL-6 decreased similarly to those of silymarin at *p* < 0.001. In addition, the lowest expressions of the two transcripts in the PBL paste and sauce extracts were observed when the insect content was 100%. For the anti-inflammatory effect, treating with the paste and sauce made of PBL results in a higher suppression of TNF-α expression in EtOH-damaged hepatocytes than treating with the paste and sauce made of GM.

### 3.6. Evaluation of Antioxidant Potential of PBL Paste and Sauce in EtOH-Damaged Hepatocytes

To examine the antioxidant potential of the PBL paste and sauce, we measured the GSH activity and SOD activity (Table 3). The GSH activity and SOD inhibition rates were 68.70% and 87.78%, respectively, in the EtOH-treated HepG2 cells as compared to the non-treated control. The GSH activity rates in the EtOH-injured HepG2 cells after pretreatment with PBP30, 50, and 100 extracts were significantly increased compared with those treated only with EtOH (68.70%). The relative GSH activity rates in the silymarin, GMP100, PBP30, PBP50, and PBP100 extract pretreatment were 160.18%, 149.84%, 149.81%, 150.40%, and 151.77%, respectively. For the pretreatment with the silymarin, GMS100, PBS30, PBS50, and PBS100 extracts, the GSH activity rates were 160.18%, 145.03%, 144.63%, 153.85%, and 154.14%, respectively (Table 3). The SOD activity (inhibition rate (%)) values in the EtOH-injured HepG2 cells after pretreatment with PBP30, 50, and 100 extracts were significantly increased compared with those treated only with EtOH (87.78%). The relative inhibition rates of SOD for the silymarin, GMP100, PBP30, PBP50, and PBP100 extracts were 161.77%, 138.38%, 149.80%, 155.76%, and 155.42%, respectively. Likewise, for the pretreatment with the silymarin, GMS100, PBS30, PBS50, and PBS100 extracts, the SOD inhibition rates were 161.77%, 145.24%, 144.05%, 155.16%, and 158.10%, respectively. In the HepG2 cells pretreated with the PBL paste and sauce extracts, the activity levels of GSH and SOD increased, as occurred with the silymarin pretreatment (160.19% and 161.77%, respectively), without significant differences (Table 3).

## 4. Discussion

In this study, fermented foods (PBL paste and sauce) were prepared using PBL larvae composed of approximately 58% protein on a dry weight basis. The hepatocyte protective effects of PBL paste and sauce, which have long been reported to have liver protection efficacy, against ethanol damage were investigated [28]. 

As a result of the analysis of 16 free amino acids in koji after fermentation, all the free amino acids except arginine and tryptophan significantly increased. However, according to previous reports, during the fermentation process of GM, only glutamic acid, glycine, aspartic acid, and proline increase rapidly [29]. Therefore, the intake of PBL fermented foods has the advantage of supplying various amino acids that are insufficient in GM fermented foods. Meanwhile, the degree of fermentation in PBL paste and sauce increased in proportion to the content of PBL, which is presumed to be due to the higher content of protein in PBL (58%) than in GM (40%) [6,29].

When the amino nitrogen level and relative protease activity was compared with pastes and sauces made of GM, it was found that the proteins were further fermented in the paste and sauce made with PBL50 and PBL100 than with GM. Furthermore, when the amino nitrogen level and relative protease activity were compared with the pastes and sauces made of 30% (TMP30: 0.369 mg/mL, 96.25%; TMS30: 2.967 mg/mL, 108.03%), 50% (TMP50: 0.408 mg/mL, 19.93%; TMS50: 3.255 mg/mL, 128.21%), and 100% (TMP100: 0.437 mg/mL, 142.6%; TMS100: 3.782 mg/mL, 13.53%) of TML [22], it was found that the proteins were further fermented in the paste and sauce made with PBL than with TML. These results are inferred, because PBL (57.86%) has a higher protein content than GM (40%) and TML (50.32%) [6,11,22].

AST and ALT hepatic enzymes have been commonly used as indices to determine hepatocyte injury severity, because they tend to increase in the blood amid necrosis and tissue destruction of hepatocytes during hepatocellular injury [30,31,32,33]. The AST and ALT levels were significantly reduced in the EtOH-injured hepatocytes pretreated with the PBL paste and sauce extracts, proportionately with the amount of PBL content. The AST and ALT values in this study were not significantly different from the levels of AST and ALT in hepatocytes damaged by EtOH pretreated with extracts of TML paste and sauce containing 30% (TMP30: 36.25 IU/L, 27.15 IU/L; TMS30: 34.73 IU/L, 26.79 IU/L), 50% (TMP50: 28.70 IU/L, 22.61 IU/L; TMS50: 29.69 IU/L, 20.94 IU/L), and 100% (TMP100: 26.46 IU/L, 19.53 IU/L; TMS100: 31.47 IU/L, 19.67 IU/L) of TML, which has previously been reported to have hepatoprotective efficacy in rats [6,33]. In addition, the reduction effect of these AST and ALT levels was superior to the traditional GMP100 and GMS100. Therefore, our results show that a daily intake of PBL paste and sauce may have a defensive effect against hepatocyte injury by EtOH.

In EtOH-injured hepatocytes pretreated with the PBL paste and sauce extracts, the expression grades of TNF-α and IL-6 were inhibited at similar levels to those of the pretreatment with silymarin, which has been known to have hepatoprotective, anti-inflammatory, antioxidant, and antiapoptosis effects [34,35,36,37]. Besides, except for the IL-6 level of PBP, it was assured that the PBL pastes and sauces showed a better TNF-α and IL-6 expression inhibitory effect than paste and sauce made by GM. Accordingly, we expect the PBL paste and sauce to have an outstanding anti-inflammatory effect on EtOH-injured hepatocytes. In addition, the inhibition of TNF-α and IL-6 expression in this study by pretreating with PBL paste and sauce extracts was considered a similar anti-inflammatory effect to that of the pretreatment with 30% (TMP30: 0.76-fold, 0.75-fold; TMS30: 0.65-fold, 0.88-fold), 50% (TMP50: 0.73-fold, 0.59-fold; TMS50: 0.84-fold, 0.67-fold), and 100% (TMP100: 0.67-fold, 0.78-fold; TMS100: 0.59-fold, 0.75-fold) TML paste and sauce extracts [22]. These results could be attributed to the fact that PBL, an animal protein, has a higher protein content and a different amino acid composition than GM, a plant protein, and has a higher liver protective effect and anti-inflammatory effect. In addition, our findings are consistent with the results that the expression levels of IL-6, TNF-α, and TGF-β decreased when orally administering *P. brevitarsis* larvae extract to rats with liver damage induced by CCl_4_ [33,38,39].

GSH activity and the SOD inhibition rate demonstrated that the pretreatment with the PBL paste and sauce extracts had a greater antioxidant effect on the EtOH-injured hepatocytes than GMP or GMS extracts. As the GSH enzyme in the liver plays a significant role in liver defense, protecting against liver damage by detoxifying many toxic compounds [40], we expected that the PBL paste and sauce might have a hepatoprotective role through antioxidant activity. This antioxidant effect is similar to that of the paste and sauce extracts made of 30% (TMP30: 151.07%, 151.75%; TMS30: 148.69%, 151.37%), 50% (TMP50: 150.31%, 156.69%; TMS50: 150.63%, 151.27%), and 100% (TMP100: 152.11%, 158.30%; TMS100: 151.74%, 151.91%) TML [22].

The above results suggest that PBL fermented food will protect the liver by inhibiting inflammation through the suppression of cytokine expression, raising GSH activity and the SOD inhibition rate, which are biomarkers of an antioxidant effect. In addition, this activity is similar to that of silymarin, used as a positive control. In the future, it will be necessary to separate a single active substance with hepatoprotective activity from PBL paste and sauce to investigate the mechanisms for hepatoprotective effects.

## 5. Conclusions

As a result of evaluating the efficacy of PBL after applying alcohol damage to hepatocytes pretreated with PBL paste and sauce extracts, it was found that PBL paste and sauce protects hepatocytes through antioxidant and anti-inflammatory effects. The degree of liver protection efficacy of the PBL paste and sauce was generally better than that of GM, similar to silymarin, and proportional to the amount of insect content included in the paste and sauce. ’Therefore, we suggest that PBL fermented food is a new food that not only has excellent taste and nutrition but also has the potential to assist in liver protection.

## Figures and Tables

**Table 1:** Amino nitrogen level and relative protease activity of each paste and sauce to evaluate PBL koji paste and sauce fermentation. The amino nitrogen level of each paste and sauce was analyzed. In addition, the protease activity of each paste and sauce was evaluated.

Samples	Amino Nitrogen (mg/mL)	Relative Protease Activity (%)
GMP 100	0.38 ± 0.01	100.00 ± 0.00
PBP 30	0.36 ± 0.02	96.73 ± 1.94
PBP 50	0.43 ± 0.01 ***	119.93 ± 2.98 ***
PBP 100	0.45 ± 0.01 ***	129.10 ± 3.44 ***
GMS 100	3.18 ± 0.13	100.00 ± 0.00
PBS 30	3.05 ± 0.10	108.03 ± 1.00
PBS 50	3.23 ± 0.18	115.19 ± 2.39 **
PBS 100	3.88 ± 0.06 ***	133.53 ± 6.76 ***

GMP100, paste made from 100% GM; PBP30, paste made from 30% PBL/ 70% GM; PBP50, paste made from 50% PBL/50% GM; PBP100, paste made from 100% PBL; GMS100, sauce made from 100% GM; PBS30, sauce made from 30% PBL/70% GM; PBS50, sauce made from 50% PBL/50% GM; PBS100, sauce made from 100% PBL. The concentrations of the paste and sauce were 400 μg/mL and 40 μl/mL, respectively. Values are the means ± standard deviations of triplicate experiments. ** *p* < 0.01 and *** *p* < 0.001 indicate a significant difference between the control and each sample made with PBL.

**Table 2:** Hepatoprotective potential of paste and sauce made from PBL koji. The AST levels in media were measured after 300 mM EtOH treatment on HepG2 cells pretreated with 95% EtOH extract of each paste and sauce. The ALT levels in media were measured after 300 mM EtOH treatment on HepG2 cells pretreated with 95% EtOH extract of each paste and sauce.

EtOH	Extract	AST (IU/L)	ALT (IU/L)
−	−	23.23 ± 3.19	25.67 ± 2.20
+	−	56.45 ± 2.20 ^##^	34.46 ± 2.02 ^##^
+	S	23.35 ± 5.38 ^**^	18.24 ± 0.76 ^***^
+	GMP 100	36.11 ± 4.12 ^**^	24.59 ± 5.99 ^**^
+	PBP 30	37.35 ± 3.20 ^**^	27.62 ± 0.49 ^**^
+	PBP 50	29.69 ± 1.87 ^**^	21.27 ± 4.54 ^**^
+	PBP 100	25.26 ± 1.43 ^**^	19.75 ± 0.88 ^***^
+	GMS 100	36.78 ± 4.23 ^**^	26.43 ± 0.79 ^**^
+	PBS 30	35.18 ± 1.44 ^**^	25.66 ± 6.48 ^**^
+	PBS 50	33.47 ± 0.11 ^**^	20.55 ± 2.29 ^***^
+	PBS 100	28.23 ± 2.97 ^**^	19.22 ± 1.51 ^***^

S, silymarin (100 μg/mL); GMP100, paste made from 100% GM; PBP30, paste made from 30% PBL/70% GM; PBP50, paste made from 50% PBL/50% GM; PBP100, paste made from 100% PBL; GMS100, sauce made from 100% GM; PBS30, sauce made from 30% PBL/70% GM; PBS50, sauce from 50% PBL/50% GM; PBS100, sauce made from 100% PBL. The concentrations of the paste and sauce were 400 μg/mL and 40 μl/mL, respectively. Values are the means ± standard deviations of triplicate experiments. ## *p* < 0.01 indicates a significant difference between the EtOH (−)/treatment (−) and the EtOH (+)/treatment (−). ** *p* < 0.01 and *** *p* < 0.001 indicate a significant difference between the EtOH (+)/treatment (−) and the EtOH (+)/treatment (+).

**Table 3:** Antioxidant potential of the paste and sauce made from PBL koji. The GSH activity was analyzed after 300 mM EtOH treatment on HepG2 cells pretreated with 95% EtOH extract of each paste and sauce. The SOD inhibition rate was investigated after 300 mM EtOH treatment on HepG2 cells pretreated with 95% EtOH extract of each paste and sauce.

EtOH	Extract	GSH Activity (%)	SOD Inhibition Rate (%)
−	−	100.00 ± 0.00	100.00 ± 0.00
+	−	68.70 ± 3.40 ^###^	87.78 ± 2.17 ^##^
+	S	160.08 ± 5.00 ^***^	161.77 ± 1.40 ^***^
+	GMP 100	149.84 ± 1.90 ^***^	138.38 ± 3.22 ^***^
+	PBP 30	149.81 ± 1.01 ^***^	149.80 ± 2.99 ^***^
+	PBP 50	150.40 ± 1.67 ^***^	155.76 ± 2.53 ^***^
+	PBP 100	151.77 ± 1.35 ^***^	155.42 ± 1.19 ^***^
+	GMS 100	145.03 ± 4.13 ^***^	145.24 ± 3.47 ^***^
+	PBS 30	144.63 ± 3.41 ^***^	144.05 ± 2.01 ^***^
+	PBS 50	153.85 ± 1.20 ^***^	155.16 ± 0.53 ^***^
+	PBS 100	154.14 ± 4.57 ^***^	158.10 ± 2.02 ^***^

S, silymarin (100 μg/mL); GMP100, paste made from 100% GM; PBP30, paste made from 30% PBL/70% GM; PBP50, paste made from 50% PBL/50% GM; PBP100, paste made from 100% PBL; GMS100, sauce made from 100% GM; PBS30, sauce made from 30% PBL/70% GM; PBS50, sauce made from 50% PBL/50% GM; PBS100, sauce made from 100% PBL. The concentrations of the paste and sauce were 400 μg/mL and 40 μl/mL, respectively. Values are the means ± standard deviations of triplicate experiments. ^##^ *p* < 0.01, ^###^ *p* < 0.001 indicates a significant difference between the EtOH (−)/treatment (−) and the EtOH (+)/treatment (−). *** *p* < 0.001 indicates a significant difference between the EtOH (+)/treatment (−) and the EtOH (+)/treatment (+).
